# Equi‐MOI ratio for rapid baculovirus‐mediated multiprotein co‐expression in insect cells integrating selenomethionine for structural studies

**DOI:** 10.1002/2211-5463.70025

**Published:** 2025-03-18

**Authors:** Andrej Bitala, Mário Benko, Marek Nemčovič, Ivana Nemčovičová

**Affiliations:** ^1^ Biomedical Research Center, Slovak Academy of Sciences Bratislava Slovakia; ^2^ Institute of Chemistry, Slovak Academy of Sciences Bratislava Slovakia

**Keywords:** baculovirus‐mediated expression vector system (BEVS), equal multiplicity of infection (equi‐MOI), insect cells expression, multigene co‐expression, recombinant protein production, selenomethionine incorporation

## Abstract

Proteins often co‐exist as multicomponent assemblies, making their co‐expression essential in recombinant production processes. The baculovirus expression vector system is commonly used to produce recombinant multiprotein complexes mostly for structural and functional studies. Although AI‐enhanced tools, such as AlphaFold, have revolutionized protein structure prediction, solving the phase problem remains the most significant challenge in X‐ray crystallography for determining entirely novel, dynamic, or complex protein structures. To address this challenge, the early incorporation of selenomethionine into native proteins during production is especially advantageous for facilitating experimental phasing. Here, we describe a fast, effective, and versatile research protocol that uniquely combines these two challenging features. The principle of this method is based on using co‐infection of several recombinant baculoviruses in so‐called equal multiplicity of infection (MOI) or equi‐MOI ratio, while at the same time, the balanced selenomethionine incorporation takes place to allow for an accelerated workflow. The delicate balance between individual conditions for producing selenomethionine‐incorporated multiprotein complexes with high efficiency has been developed over several years of studying protein complexes; therefore, many useful tips and tricks are provided as well. Moreover, this protocol is straightforward to implement in any wet lab.

## Graphical abstract

This study presents a versatile protocol for producing selenomethionine‐labeled multiprotein complexes, addressing challenges in structural biology such as experimental phasing in X‐ray crystallography. Its efficiency and adaptability make it valuable for researchers studying *de novo*, dynamic, or complex protein structures, promoting advancements in structure‐functional studies across biomedical and biotechnological fields.

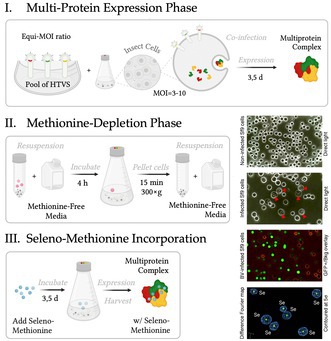

**Abbreviations**
AcMNPV: *Autographa californica* multiple nucleopolyhedrovirus
AI: artificial intelligence
BAC: bacterial artificial chromosome‐derived baculovirus
BEVS: baculovirus expression vector system
biGBac: tool for assembling and expressing multiple genes using baculoviruses and Golden Gate cloning technology
Bsu36I: type II restriction endonuclease isolated from the bacterium *Bacillus subtilis*
CD: cluster of differentiation
CD155: poliovirus receptor
DNA: deoxyribonucleic acid
*E. coli*
: bacterium *Escherichia coli*
EPDA: endpoint dilution assay
equi‐MOI ratio: balanced or equal MOI ratio
FBS: fetal bovine serum
GFP: green fluorescent protein
HCMV: human cytomegalovirus
HTVS: high titer virus stock
KO: knockout – modification in a particular gene
MAD: multi‐wavelength anomalous dispersion
mCherry: monomeric Cherry (a red fluorescent protein)
MOI: multiplicity of infection
ORF: open reading frame
P1: first passage of virus or low titer virus stock
qPCR: quantitative polymerase chain reaction
rpm: rotations per minute
RT: room temperature
SAD: single‐wavelength anomalous dispersion
SDS/PAGE: sodium dodecyl sulfate–polyacrylamide gel electrophoresis
SeMet: selenomethionine
Sf9 cells: cell line derived from *Spodoptera frugiperda*
TR2: TNF‐related apoptosis‐inducing ligand receptor 2
UL141: viral immunomodulatory protein encoded by HCMV

The production of a homogeneous protein sample in sufficient quantities is an essential prerequisite not only for structural investigations but also represents a rate‐limiting step for many functional studies and drug design. In the living cell, a large fraction of proteins co‐exists as multicomponent assemblies. As many of them cannot be obtained from endogenous sources, the recombinant expression is required to overcome this bottleneck. The baculovirus technology was developed in the eighties [1, 2]; however, the breakthroughs came 10 years later [3, 4, 5, 6, 7, 8] and since then it serves as a core for a number of commercial vectors (see Materials section). The baculovirus expression vector system (BEVS) has become a widely used tool to produce recombinant proteins [9, 10, 11, 12, 13], especially those requiring complex post‐translational modifications. BEVS is particularly advantageous for studying protein complexes and interactions. These proteins can be expressed from multiple baculoviruses (via co‐infection), each carrying a single monocistronic foreign gene, or from a single polycistronic baculovirus containing multiple foreign genes. Some studies have highlighted rapid gene assembly methods, such as biGBac, for the efficient expression of large multisubunit protein complexes by using polycistronic vectors [8]. While the use of multigene vectors is well‐established, the application of monocistronic vectors for producing multiprotein assemblies remains less explored, despite its significant potential for such purposes. Moreover, incorporating selenomethionine (SeMet) into native proteins early in the production process significantly enhances the system's utility for structural studies [14, 15]. SeMet is an analog of methionine, where the sulfur atom is replaced with selenium during protein synthesis. This substitution is particularly useful in X‐ray crystallography, as selenium atoms provide unique anomalous scattering properties that facilitate phase determination through techniques such as multi‐wavelength anomalous dispersion (MAD) or single‐wavelength anomalous dispersion (SAD). While AI‐based tools like AlphaFold [16] have revolutionized protein structure prediction, the phase problem remains to be a critical bottleneck in X‐ray crystallography, especially for proteins with unusual or novel folds (e.g., viral proteins, or proteins from unknown organism); multiprotein complexes or macromolecular assemblies (e.g., ribosomes, viral capsid glycoproteins, or protein‐DNA complexes); proteins with bound ligands (e.g., drugs, cofactors, phosphorylation, or glycosylation) and dynamic structures. Therefore, experimental phasing techniques, such as SAD and MAD, are essential for resolving the phase problem in the determination of *de novo* protein structures. However, achieving efficient SeMet incorporation in insect cell systems also poses several challenges [15, 17, 18, 19]. Optimizing the concentration of SeMet in the cell culture and ensuring that it is effectively incorporated during protein synthesis without causing toxicity or misfolding is critical for the success of this approach. In this study, we present a fast, effective, and versatile protocol that uniquely combines these challenging features. Considering also that initial infectivity is a reliable indicator of overall protein complex production, as described in detail in [7], we have established and verified a balanced multiplicity of infection (MOI) ratio, termed the equi‐MOI ratio. This method involves co‐infecting cells with several monocistronic baculoviruses at an equi‐MOI ratio while simultaneously ensuring balanced SeMet incorporation. This approach enables an accelerated workflow for the efficient production of protein complexes. The experimental procedures reported were developed over years of producing various viral and endogenous protein complexes for our structural studies, including HCMV UL141 protein in complex with TRAIL death receptor 2 or in complex with HCMV US2 and CD155; and the HCMV UL144 glycoprotein in complex with immunoreceptors BTLA and CD160; among others [20, 21, 22, 23, 24, 25, 26, 27]. This protocol is well‐suited for the rapid SeMet‐incorporated multiprotein co‐expression of multiprotein complexes and can be easily implemented in every wet lab.

## Materials

Reagents and materials required to successfully perform this protocol can be purchased from many vendors either as separate products or as complete kits. Below, we provide a concise list of all key reagents along with possible alternatives that were tested to optimize this protocol.

### Competent transfer vectors

Several versatile transfer vectors have been constructed that permit the insertion of foreign genes between polyhedrin flanking sequences and allow the co‐transfection along with wild‐type viral DNA into insect cells. According to different requirements, a wide range of combinations is available, e.g. with single or multiple promoters for single or simultaneous expression with various tags, or secretory protein with signal peptide.

1. pBacPAK™8 transfer vector (Cat. No. 631402; Takara Bio, Kusatsu, Japan)
2. pAcGP67A™ baculovirus transfer vector (Cat. No. TVC2019; Creative BioMart, Shirley, NY, USA)
3. pVL1393™ vector (Cat. No. B1; AB Vector, San Diego, CA, USA)
4. pOET1.1™ transfer plasmid (Cat. No. 200101; Oxford Expression Technologies, Oxford, UK)

### Baculovirus DNA

The bacmid BAC10:KO1629 developed by [28] is the original source of baculovirus DNA. It consists of the wild‐type AcMNPV genome in which part of ORF1629 that is essential for virus replication has been replaced by a low copy bacterial replicon and resistance markers that are surrounded by Bsu36I restriction sites used for DNA linearization. ORF1629 is rescued after recombination with the transfer vector. Genetically optimized, ready‐to‐use linearized baculovirus DNA, often including features such as fluorescent markers, is commercially available from several suppliers.

1. ProGreen™ viral DNA (Cat. No. A1; AB Vector, San Diego, CA, USA)
2. BestBac™ linearized baculovirus DNA (Cat. No. 91‐001; Expression System, Davis, CA, USA)
3. BacPAK™6 viral DNA (Cat. No. 631402; Takara Bio, Kusatsu, Japan)
4. flashBAC™ ULTRA baculovirus DNA (Cat. No. 100300; Oxford Expression Technologies, Oxford, UK)

### Lipidic transfection reagents

For the initial transfection, several reagents can be used. In our hands, the transfection with any of them works with comparable efficiency.

1. Expres^2^ TR™ transfection reagent (Cat. No. 91‐100; Expression System, Davis, CA, USA)
2. ProFectin™ reagent (Cat. No. T10; AB Vector, San Diego, CA, USA)
3. CellFectin II™ reagent (Cat. No. KTR1027; Creative BioMart, Shirley, NY, USA)
4. BacFectin™ (Cat. No. 631402; Takara Bio, Kusatsu, Japan)
5. baculoFECTIN II™ transfection reagent (Cat. No. 300105; Oxford Expression Technologies, Oxford, UK)

### Special chemicals

1. L(+)‐Selenomethionine > 99%, C_5_H_11_NO_2_Se, Mw 196.12 (Cat. No. 259960010, CAS No. 3211‐76‐5; Acros Organics, Geel, Belgium)
2. Cytiva HyClone™ dialyzed Fetal Bovine Serum (FBS) (Cat. No. SH30088.03; Cytiva, Marlborough, MA, USA)
3. Baculovirus Titering Kit (Cat. No. 97‐101; Expression System, USA) or BacPAK p35 ELISA Titering Kit (Cat. No. 631477; Takara Bio, Kusatsu, Japan)

### Insect cells

1. Sf9 cells from *Spodoptera frugiperda* (Cat. No. 94‐001F; Expression System, Davis, CA, USA)
2. Sf21 cells from *Spodoptera frugiperda* (Cat. No. 11497013; Thermo Fisher Scientific, Waltham, MA, USA)
3. Tni cells from *Trichoplusia ni* (Cat. No. 94‐002F; Expression System, Davis, CA, USA)
4. High Five™ (Hi5) cells from *Trichoplusia ni* (BTI‐TN‐5B1‐4, Cat. No. B85502; Invitrogen, Carlsbad, CA, USA)

### Antibiotics

1. Penicillin–Streptomycin (Cat. No. 17‐603E; Lonza, Basel, Switzerland)
2. Gentamicin Sulfate (Cat. No. 17‐518L; Lonza, Basel, Switzerland)

### Media for insect cells and their compatibility

The selection of media is recommended based on its compatibility with other critical reagents, such as the transfer vector and viral DNA. Among the four tested media, the first three demonstrated full compatibility with all the listed vectors, viral DNAs, and other reagents, whereas the last medium exhibited reduced compatibility but remained suitable for use with reagents from the same manufacturer.

1. Insect‐XPRESS™ protein‐free with L‐glutamine (Cat. No. 12‐730Q; Lonza, Basel, Switzerland)
2. ESF 921Δ™ Delta series methionine deficient (Cat. No. 96‐200; Expression Systems, Davis, CA, USA)
3. Sf‐900 II SFM™ (Cat. No. MED1211; Creative BioMart, Shirley, NY, USA)
4. Transfection Medium™ complement (Cat. No. 95020‐100; Expression System, Davis, CA, USA)

## Methods

### Intracellular homologous recombination

Homologous recombination takes place in intracellular compartments, and it is a commonly used method to insert the gene of interest into baculovirus DNA in BEVS. The most common expression systems (see Materials section) apply the so‐called rescue strategy to solve this problem. Vectors such as Bac‐to‐Bac™ (Invitrogen, Carlsbad, CA, USA) are based on the other recombination technology in *E. coli*, but this is not intended in this protocol. The viral DNA is engineered with a lethal deletion, and it is pre‐linearized at three Bsu36I restriction enzyme recognition sites. A part of the essential ORF1629 is cut off by the enzyme; thus, the linear viral DNA can neither self‐link nor survive in a non‐recombined form. Only when the viral DNA is co‐transfected into the insect cell and homologously recombined with the transfer plasmid which contains the segment of the gene of interest can the recombinant virus DNA survive in the host insect cell.

### Preparation of recombinant transfer vector (Step 1)

**Fig. 1:**
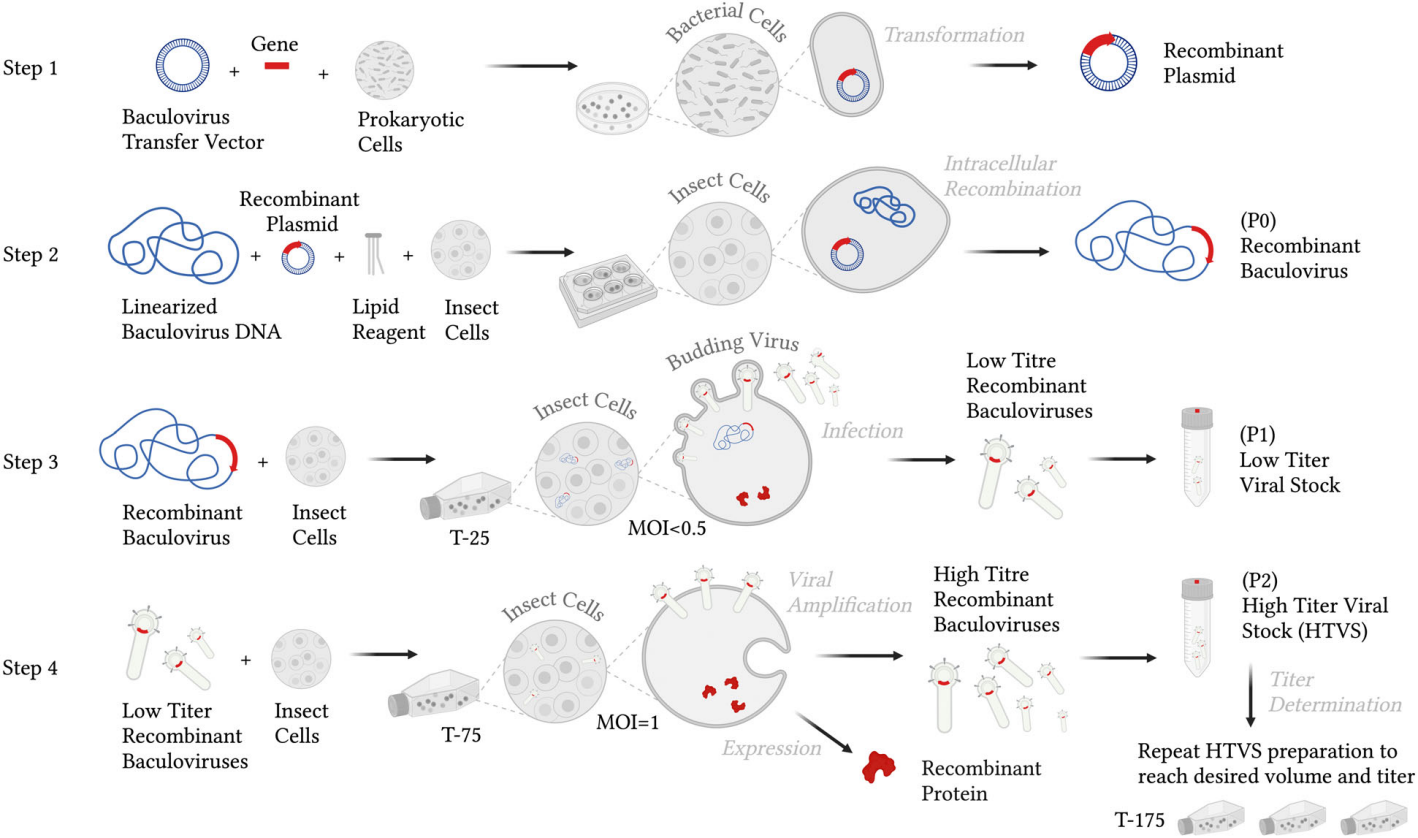
The simplified scheme showing the protocol for optimized baculovirus‐mediated expression in insect cells. The procedures shown here are as follows: preparation of recombinant transfer vector (Step 1), generation of recombinant baculoviruses (Step 2), virus amplification to obtain low titer virus stock (Step 3), or high titer virus stock (Step 4).

The mature ectodomain of the gene of interest, along with selected functional tags, is typically PCR‐amplified from the source DNA and then cloned downstream of the secretion signal sequence in a specific baculovirus transfer vector. Recombinant plasmids are subsequently amplified in standard bacterial strains and maintained under sterile conditions. Conventional cloning methods can be applied to generate recombinant transfer plasmids (Fig. 1/Step 1).

### Generation of recombinant baculoviruses (Step 2)

1. Seed 0.8 × 10^6^ insect cells from a confluent suspension culture into a 6‐well tissue culture plate. Add protein‐free medium in each well to a total volume of 3 mL. In a typical experiment, include one well that contains only cells, one that contains only medium, and a positive fluorescent transfection control. For each, seed two wells. Allow cells to incubate for 15 min at 27 °C.
2. For each construct, prepare transfection complexes by diluting 0.5 μg of linearized viral DNA and 2 μg of transfer plasmid containing the gene of interest (centrifuged at high speed) in 250 μL insect cell protein‐free medium. Dilute 20 μL of transfection reagent in 250 μL of medium in a separate tube.
3. Add the diluted transfection reagent to the DNA solution (respect the order of addition), vortex for 20 s, and incubate at room temperature (RT) for 20 min, protected from light.
4. Add 0.5 mL of medium to the transfectant‐DNA suspension and use it to replace the supernatant from seeded cells. Apply dropwise to the cells. Rock back and forth the plate horizontally every 20–30 min. Incubate for 4 h at RT protected from light. Replace the supernatant with 3 mL of fresh protein‐free medium, including antibiotics. Incubate for a maximum of 5 days at 27 °C.
5. Pellet cells (10 min at 1000 ***g***) and carefully collect the supernatant and store at 4 °C protected from light. This is the passage 0 (P0) of recombinant virus. The P0 stock can be used directly to test expression of the desired proteins by using western blot or could be concomitantly amplified for production (Fig. 1/Step 2). See also paragraph ‘Tips & Tricks’.

### Virus amplification (Step 3–4)

1. Seed 2 × 10^6^ insect cells from a confluent suspension culture into a T‐25 tissue culture flask. Add protein‐free medium to a total volume of 5 mL. Infect at MOI of 0.5 or below with a small volume (roughly ~500 μL) of P0 virus and incubate for 5 days at 27 °C. The volume of virus depends on the titer of your virus stock and thus on transfection efficiency.
2. Transfer the suspension into a fresh 15 mL tube and spin for 10 min at 1000 ***g***. Collect the supernatant into a fresh tube and supplement with 10% FBS if a serum‐free medium was used and store at 4 °C protected from light. This is the (P1) low titer virus stock reaching approximately ~10^3^–10^6^ pfu·mL^−1^ (Fig. 1/Step 3). Keep the pellet resuspended in small amount of fresh medium for further analysis. If large volume of virus is required, amplify P1 to obtain P2 (and eventually P3). See also paragraph ‘Tips & Tricks’.
3. For amplification, seed 3 × 10^6^ insect cells from a confluent suspension stock culture into a T‐75 tissue culture flask. Add medium to a total volume of 15 mL. Infect at an MOI of 1 (or below) with P1 virus (roughly ~1.5 mL) and incubate for 5 days at 27 °C. The volume of virus depends on the titer of your virus stock and thus on transfection efficiency.
4. Transfer the suspension into a fresh 50 mL tube and spin for 10 min at 1000 ***g***. Collect the supernatant into a fresh tube and supplement with 5% FBS if a serum‐free medium was used and store at 4 °C protected from light. This is the (P2) high titer virus stock (HTVS) reaching approximately ~10^7^–10^9^ pfu·mL^−1^ (Fig. 1/Step 4).
5. If more virus is needed for protein production, repeat the HTVS preparation with higher amplitude in parallel flasks. For example, infect 1 × 10^7^ cells with P2 virus at MOI of 1 (roughly ~100 μL of P2) in 50 mL of media in T‐175 flask. HTVS are used for infection of cells at optimal multiplicity of infection resulting in maximum protein production. See also paragraph ‘Tips & Tricks’.

### Determination of viral titer

**Fig. 2:**
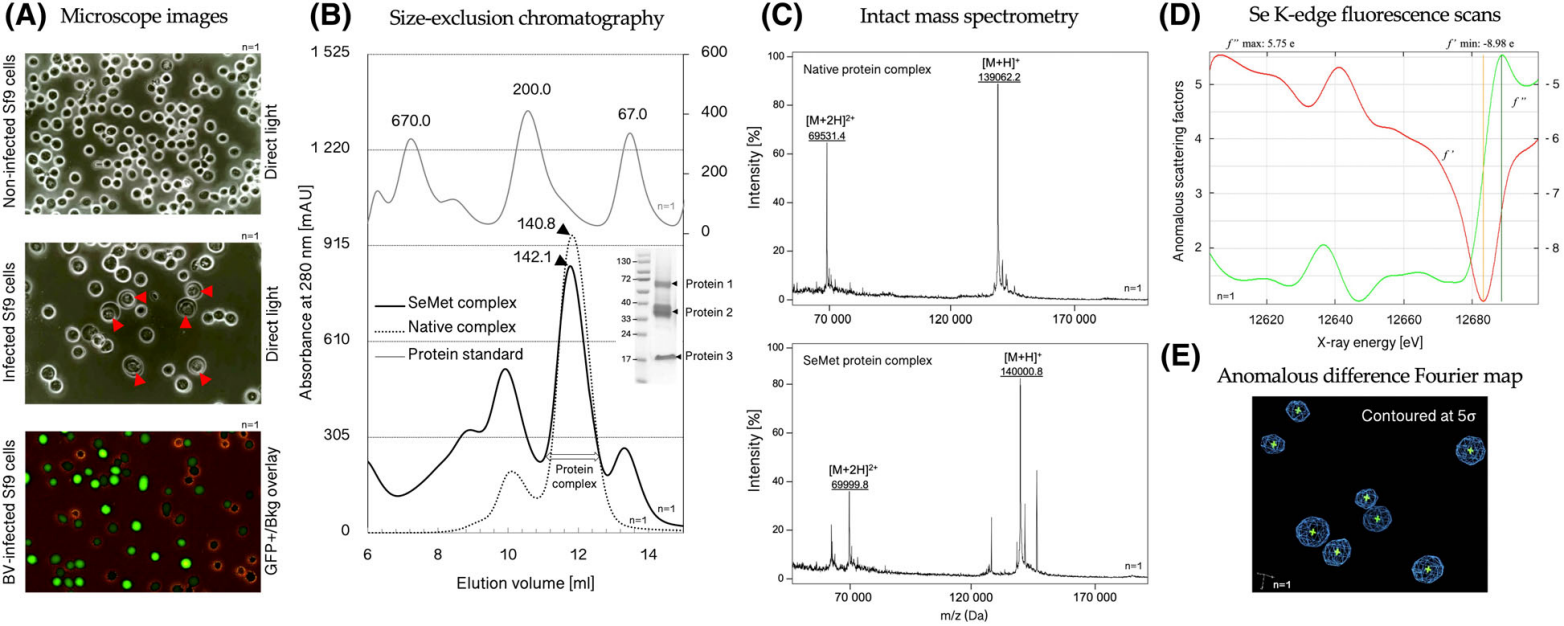
(A) Representative microscopy images showing differences between non‐infected cell culture (top panel) and baculovirus‐infected cells (two bottom panels). Red arrows in the middle panel indicate visible signs of successful baculovirus (BV) infection. In the bottom panel, the overlay of GFP‐expressing cells (green fluorescent protein, GFP) against the background further confirms successful baculovirus transfection in the cell culture expressing recombinant protein complex. (B) Efficient production of the three‐protein complex (as indicated in the figure) was achieved through co‐expression in BV‐Sf9 cells. Protein expression levels were relatively high (~6.1 mg of purified protein complex) given the culture volume (1000 mL). Size‐exclusion chromatography (SEC) confirmed the formation of the three‐protein complex, with chromatograms of the SeMet‐incorporated (bold line) and native protein complex (dotted line) shown side by side. Molecular size standards (with indicated molecular weights in kDa) are provided for reference in the top panel. Gel electrophoresis (SDS‐PAGE) analysis of the purified protein complex confirms that each component migrates at its expected molecular weight. (C) Mass spectrometry analysis was performed to determine the intact masses of the SeMet‐substituted (bottom panel) and native protein complexes (top panel). The differently charged peaks for both protein complexes are shown, with *m*/*z* values of 69 531.4 and 139 062.2 for the native complex, and 69 999.8 and 140 000.8 for the SeMet‐incorporated complex. The observed mass shift from 139 062.2 Da to 140 000.8 Da upon SeMet incorporation (a difference of 938.6 Da) corresponds to an incorporation efficiency exceeding 96%. (D) The selenium‐labeled protein complex was successfully crystallized and Se K‐edge fluorescence scans conducted at the synchrotron confirmed Se incorporation. The real (f′) and imaginary (f″) components of anomalous scattering are plotted as a function of incident photon energy. (E) The Se anomalous difference Fourier map, calculated between 6.1 and 2.4 Å resolution and contoured at 5σ, is shown in blue. Nineteen out of twenty SeMet residues were identified in the map, with the SeMet at position 1 located in a disordered region of the protein. For all presented plots, we observed high reproducibility across at least three independent biological experiments (*n* = 3–5). A representative replicate (*n* = 1) is shown, as indicated next to the curve.

The virus titer could be exactly measured by classical plaque assay, flow cytometry, or qPCR; however, for its estimation, the endpoint dilution assay (EPDA) could be used instead. Alternatively, the baculovirus titering kit (see Materials section) could be used for viral titer estimation. EPDA only requires careful observation and handling while measuring the amount of virus required to infect cultured cells, with focus on the highest dilution at which the virus still causes an observable effect. To perform the EPDA, follow these steps:

1. Seed 1 × 10^3^ insect cells from a confluent suspension culture into a 6‐well tissue culture plate. Add protein‐free medium to each well to a total volume of 2 mL. Include one well that contains only cells, as an uninfected control. For each virus dilution, seed two wells. Allow the cells to incubate for 15 min at 27 °C.
2. Prepare the 10‐fold serial dilutions of the virus in the culture medium, or the cells could be directly inoculated with 100, 10, and 1 μL aliquots of the collected supernatant containing virus (i.e., P0‐P2). Incubate cells for 3 days at 27 °C.
3. Cells are visually inspected for signs of infection (Fig. 2A) however, it may be difficult to identify infected cells as signs of infection are not always visually apparent, particularly if the transfection efficiency is low. The visual comparison between dilutions is used to ascertain how successful was infection. If the transfection supernatant shows a 10‐fold decrease in the number of infected cells between dilutions, you should amplify the virus once or twice more. See also paragraph ‘Tips & Tricks’.

### Native multiprotein expression (Step 5a)

**Fig. 3:**
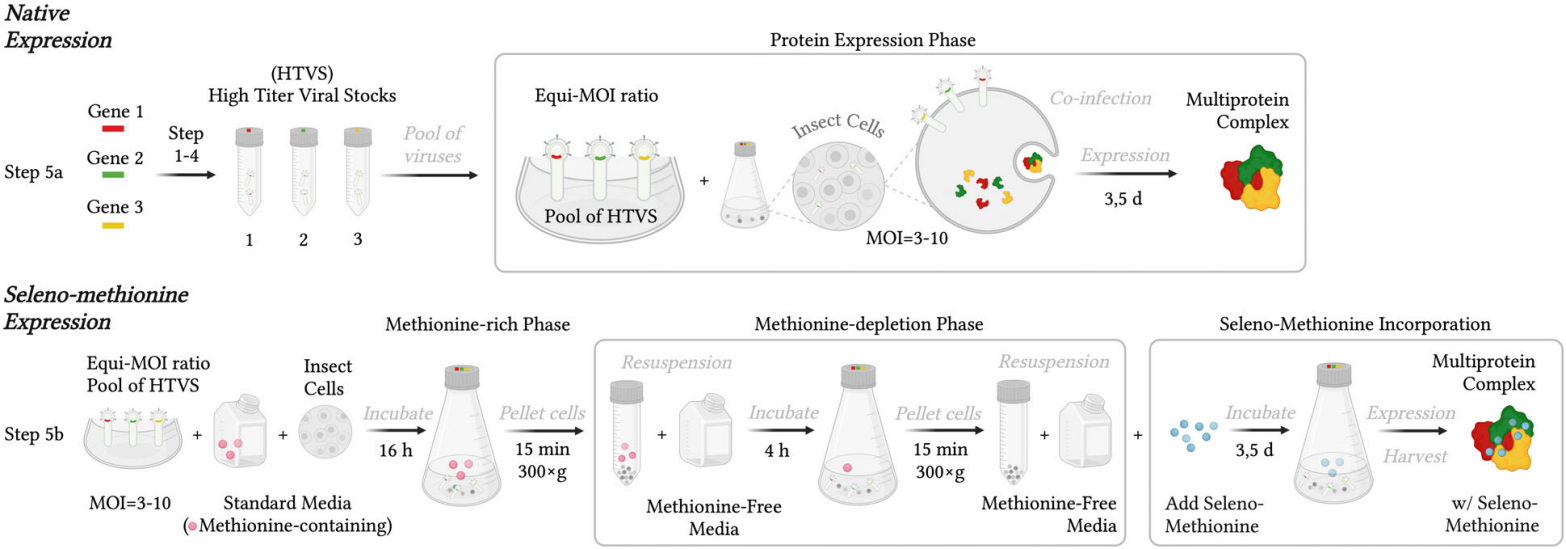
The simplified scheme showing the protocol for rapid baculovirus‐mediated equi‐MOI (equal multiplicity of infection) multiprotein co‐expression in insect cells integrating selenomethionine for native structural studies. The procedures shown here are as follows: native multiprotein expression (Step 5a) and selenomethionine (SeMet) multiprotein expression (Step 5b).

1. Prepare one 2 L Erlenmeyer flask (or several, if needed) containing 1000 mL of cell suspension at a density of 2 × 10^6^ cells·mL^−1^ in the exponential growth phase. Use standard protein‐free media.
2. Infect the cell culture at MOI 3–10 with a heterologous virus that was freshly pooled from several P2 viruses, each at ~ MOI 3, to maintain an equi‐MOI ratio (roughly ~40 mL each). The volume of virus depends on the titer of your virus stock and thus on transfection efficiency. Incubate the cell culture at 27 °C with low agitation (max. 150 rpm) for 3.5 days (approx. 84 h).
3. Transfer the suspension into several centrifugal tubes and spin for 10 min at 1000 ***g***. Collect the supernatant containing secreted proteins into a fresh 50 mL tubes and spin for an additional 30 min at high speed (20 000 ***g***) 4 °C to remove any residual cell debris. If protein is not aimed to be secreted into media (depends on protein properties and vector usage), collect the cell pellet and proceed with cell lysis and isolation.
4. Harvested proteins secreted into the culture media should immediately undergo initial purification (Fig. 2B) or concentration steps and should not be stored at this stage to preserve their integrity and functionality (Fig. 3/Step 5a). See also paragraph ‘Tips & Tricks’.

### Selenomethionine multiprotein expression (Step 5b)

1. Repeat procedure 1–2 of Step 5a but incubate only for 16 h (min. 8–36 h).
2. To achieve depletion of methionine from intracellular pools, gently spin down the suspension for 15 min at 300 ***g***. Discard the supernatant and gently resuspend the cells in an equal volume of methionine‐free medium supplemented with antibiotics. Incubate the suspension with low agitation at 27 °C for 4 h (min. 4–12 h).
3. Gently spin down the suspension again for 15 min at 300 ***g***. Discard the supernatant and gently resuspend the cells in an equal volume of methionine‐free medium supplemented with antibiotics.
4. Add 50 mg/L of selenomethionine (SeMet). The critical point of SeMet addition is within the first 16–20 h following viral infection, as the protein expression begins at that time. Expression of the protein complex was continued for 48–96 h post‐infection. Total time of expression is ideally 3.5 days at 27 °C with low agitation (max. 150 rpm).
5. Transfer the suspension into several centrifugal tubes and spin for 10 min at 1000 ***g***. Collect the supernatant containing secreted proteins into a fresh 50 mL tubes and spin for an additional 30 min at high speed (20 000 ***g***) 4 °C to remove any residual cell debris. If protein is not aimed to be secreted into media (depends on protein properties and vector usage), collect the cell pellet and proceed with cell lysis and isolation.
6. Harvested proteins secreted into the culture media should immediately undergo initial purification (Fig. 2B) or concentration steps and should not be stored at this stage to preserve their integrity and functionality (Fig. 3/Step 5b). See also paragraph ‘Tips & Tricks’.

### Storing viruses

Virus stock (especially P2) can be safely stored for years, but it should be protected from light and kept at 4 °C. An alternative option is to store frozen aliquots of baculovirus‐infected insect cells with a final density of 1 × 10^7^ cells·mL^−1^ that can be stored in liquid nitrogen for an indefinite time. The integrity of the recombinant virus can be checked by various methods, including PCR.

## Tips & Tricks

1. For some protein complexes, the stoichiometry (the ratio of different subunits) may not be 1:1. Adjustments to the equi‐MOI ratio (in Step 5a/5b) might be necessary to reflect the natural stoichiometry of the complex.
2. After 48 h post‐infection (Step 2), the most, if not all, of the cells should show substantial swelling, and proliferation arrest should be observed. The culture should be harvested when cells have been infected for several days, ideally 24 h after cells have stopped dividing, or when cells in the positive control express the fluorescent protein. This control typically involves the co‐transfection of cells with a plasmid encoding a fluorescent protein (e.g., GFP or mCherry) alongside the plasmid of interest. The fluorescent protein acts as a marker, enabling visualization of successfully transfected cells under a fluorescence microscope. This allows for the direct evaluation of transfection efficiency (Fig. 2A).
3. Rough estimation of virus volume (in Step 3–4). For amplification, the MOI should be 0.1–0.5. Ideally, the titer of the P0 stock should be determined experimentally. However, assuming a cell concentration of 0.4 × 10^6^ cells·mL^−1^ and a titer of the P0 stock of 2 × 10^6^ pfu·mL^−1^, then an MOI of 0.1–0.5 would correspond to 2–10% (virus volume/culture volume percentage). For example, add between 100 and 500 μL of virus stock to a 5 mL culture in a T‐25 flask.
4. If the volume of virus (in Step 2–3) added to the culture was adequate, cells should look healthy and should have doubled after 24 h post‐infection. At this time, infected cells should be releasing budded virus into the medium to infect other cells. If too much virus was added, signs of infection are visible: cells swell (size can increase up to 20–30%), stop dividing, and appear uniformly rounded with enlarged nuclei. Restart with less virus.
5. P1 virus is sufficient for initial protein expression studies. If large volumes of virus are required for large‐scale protein expression, amplify P1 to obtain P2 (and eventually P3). Remember, a 48 h post‐infection usually yields a 2‐log amplification.
6. While performing EPDA, the cell viability can be evaluated with Trypan blue. Nonviable cells will take up blue color. Healthy cultures should contain more than 97% of unstained viable cells. An automated counter can be used to provide cell size distribution, which is an indicator of cell infection.
7. Detailed monitoring of virus amplification (in Step 2–5) is not always possible when a large number of viruses are needed simultaneously. For this reason, the protocol contains given volumes of viruses P0–P3 to simplify the process.
8. A successful transfection should result in uniformly large, infected cells in all experimental wells during EPDA. If only the 100 and 10 μL wells seem to have infected cells and the 1 μL well looks more like the control, then the titer of your virus supernatant is low. Amplify the virus an additional time before you proceed with protein production.
9. When co‐expressing multiple proteins in the BEVS (in Step 5a/5b), the equi‐MOI ratio is particularly important. By using an equal MOI for each recombinant baculovirus, the balanced expression levels of all target proteins could be achieved. This balance is crucial for the proper assembly and functionality of protein complexes. For example, if three proteins are to be co‐expressed, each recombinant baculovirus encoding one of these proteins would be added at the same MOI. If the chosen MOI is 3, each of the three baculoviruses would be added to the culture at an MOI of 3, resulting in a combined MOI of 9. This approach helps to ensure that each cell receives approximately the same number of virus particles for each gene of interest, promoting uniform expression (Fig. 2B).
10. High MOIs can sometimes be toxic to cells. It is important to find a balance that maximizes protein expression while maintaining cell health.
11. The medium in any step could be supplemented with 5–10% (v/v) heat‐inactivated FBS when necessary.
12. Respect the incubation time recommended for use of transfection reagent (in Step 2) as extended incubation may lead to the formation of large and difficult‐to‐transfect DNA/transfection complexes. Carefully follow the manufacturer's instructions for procedures and the choice of compatible medium.
13. Maximum expression is usually observed between 30 and 84 h for secreted proteins and between 48 and 96 h post‐infection for non‐secreted proteins.
14. Secreted proteins in conditioned media should not be stored for long periods due to the risk of degradation or modifications (such as proteolysis) by extracellular enzymes, which can compromise protein quality. In contrast, intracellular proteins that are collected from the cell pellet are generally more stable and can be stored for longer periods, as they are less exposed to such degradation pathways. Therefore, immediate purification or concentration is recommended to ensure protein integrity and functional yield.
15. A protocol is described to produce soluble recombinant protein complexes with a very high SeMet occupancy rate (approx. 80–100%). The success of SeMet substitution often depends on the specific protein and its natural methionine content. The SeMet incorporation can be monitored by mass spectrometry by comparing the mass shifts in the intact protein (Fig. 2C) or the protein's peptides. Thus, the incorporation of SeMet can be confirmed due to the increase in mass corresponding to the replacement of sulfur in methionine with selenium, resulting in an approximate mass increase of 46.93 Da per substitution. Additionally, when crystals are available, Se K‐edge fluorescence scans and difference Fourier maps can be utilized to detect anomalous selenium signals (Fig. 2D,E).
16. Addition of specific cell culture antibiotics can be useful when it is necessary to face a contamination. For example, during the methionine‐depletion phase, etc. A typical concentration of 5–25 U·mL^−1^ of penicillin and/or 7 μg·mL^−1^ of streptomycin and/or 5–50 μg·mL^−1^ of gentamicin can be added to cell culture to prevent contamination.

## Conflict of interest

The authors declare that they have no conflicts of interest with the contents of this article.

## Author contributions

IN, AB, and MB developed and tested the protocol in various settings. MN contributed special reagents and analysis. IN and MN wrote the manuscript. All authors edited the final version.

## Data Availability

All research data are contained within the manuscript. Further information and requests for resources, data, and reagents should be directed to and will be fulfilled by the Lead Contact, Ivana Nemčovičová (viruivka@savba.sk). Cells, constructs, or special reagents are available upon request, subject to our institutional and material transfer agreements.

## References

[1] Smith GE , Summers MD and Fraser MJ (1983) Production of human beta interferon in insect cells infected with a baculovirus expression vector. Mol Cell Biol 3, 2156–2165. 6318086 10.1128/mcb.3.12.2156-2165.1983 PMC370086

[2] Pennock GD , Shoemaker C and Miller LK (1984) Strong and regulated expression of escherichia coli beta‐galactosidase in insect cells with a baculovirus vector. Mol Cell Biol 4, 399–406. 6325875 10.1128/mcb.4.3.399-406.1984 PMC368716

[3] Kitts PA and Possee RD (1993) A method for producing recombinant baculovirus expression vectors at high frequency. Biotechniques 14, 810–817. 8512707

[4] Gierse JK , Luckow VA , Askonas LJ , Duffin KL , Aykent S , Bild GS , Rodi CP , Sullivan PM , Bourner MJ and Kimack NM (1993) High‐level expression and purification of human leukotriene A4 hydrolase from insect cells infected with a baculovirus vector. Protein Expr Purif 4, 358–366. 8251746 10.1006/prep.1993.1047

[5] Luckow VA (1993) Baculovirus systems for the expression of human gene products. Curr Opin Biotechnol 4, 564–572. 7764207 10.1016/0958-1669(93)90078-b

[6] Luckow VA , Lee SC , Barry GF and Olins PO (1993) Efficient generation of infectious recombinant baculoviruses by site‐specific transposon‐mediated insertion of foreign genes into a baculovirus genome propagated in *Escherichia coli* . J Virol 67, 4566–4579. 8392598 10.1128/jvi.67.8.4566-4579.1993 PMC237841

[7] Imasaki T , Wenzel S , Yamada K , Bryant ML and Takagi Y (2018) Titer estimation for quality control (TEQC) method: a practical approach for optimal production of protein complexes using the baculovirus expression vector system. PLoS One 13, e0195356. 29614134 10.1371/journal.pone.0195356 PMC5882171

[8] Weissmann F , Petzold G , VanderLinden R , Huis In't Veld PJ , Brown NG , Lampert F , Westermann S , Stark H , Schulman BA and Peters JM (2016) BigBAC enables rapid gene assembly for the expression of large multisubunit protein complexes. Proc Natl Acad Sci USA 113, e2564–e2569. 27114506 10.1073/pnas.1604935113 PMC4868461

[9] Sokolenko S , George S , Wagner A , Tuladhar A , Andrich JM and Aucoin MG (2012) Co‐expression vs. co‐infection using baculovirus expression vectors in insect cell culture: benefits and drawbacks. Biotechnol Adv 30, 766–781. 22297133 10.1016/j.biotechadv.2012.01.009 PMC7132753

[10] Scholz J and Suppmann S (2017) A new single‐step protocol for rapid baculovirus‐driven protein production in insect cells. BMC Biotechnol 17, 1–9. 29145860 10.1186/s12896-017-0400-3 PMC5689143

[11] Kolesnikova O , Zachayus A , Pichard S , Osz J , Rochel N , Rossolillo P , Kolb‐Cheynel I , Troffer‐Charlier N , Compe E , Bensaude O *et al*. (2022) HR‐BAC, a toolbox based on homologous recombination for expression, screening and production of multiprotein complexes using the baculovirus expression system. Sci Rep 12, 2030. 35132103 10.1038/s41598-021-04715-5 PMC8821708

[12] Berger I , Fitzgerald DJ and Richmond TJ (2004) Baculovirus expression system for heterologous multiprotein complexes. Nat Biotechnol 22, 1583–1587. 15568020 10.1038/nbt1036

[13] Berger I and Poterszman A (2015) Baculovirus expression: old dog, new tricks. Bioengineered 6, 316–322. 26488462 10.1080/21655979.2015.1104433 PMC4825837

[14] Doublie S (1997) Preparation of selenomethionyl proteins for phase determination. Methods Enzymol 276, 523–530. 9048379

[15] Doublie S (2007) Production of selenomethionyl proteins in prokaryotic and eukaryotic expression systems. Methods Mol Biol 363, 91–108. 17272838 10.1007/978-1-59745-209-0_5

[16] Jumper J , Evans R , Pritzel A , Green T , Figurnov M , Ronneberger O , Tunyasuvunakool K , Bates R , Zidek A , Potapenko A *et al*. (2021) Highly accurate protein structure prediction with Alphafold. Nature 596, 583–589. 34265844 10.1038/s41586-021-03819-2 PMC8371605

[17] Cronin CN , Lim KB and Rogers J (2007) Production of selenomethionyl‐derivatized proteins in baculovirus‐infected insect cells. Protein Sci 16, 2023–2029. 17660253 10.1110/ps.072931407 PMC2206972

[18] Barton WA , Tzvetkova‐Robev D , Erdjument‐Bromage H , Tempst P and Nikolov DB (2006) Highly efficient selenomethionine labeling of recombinant proteins produced in mammalian cells. Protein Sci 15, 2008–2013. 16823037 10.1110/ps.062244206 PMC2242577

[19] Wenzel S , Imasaki T and Takagi Y (2019) A practical method for efficient and optimal production of seleno‐methionine‐labeled recombinant protein complexes in the insect cells. Protein Sci 28, 808–822. 30663186 10.1002/pro.3575 PMC6423997

[20] Bitra A , Nemčovičová I , Picarda G , Doukov T , Wang J , Benedict CA and Zajonc DM (2019) Structure of human cytomegalovirus UL144, an HVEM orthologue, bound to the B and T cell lymphocyte attenuator. J Biol Chem 294, 10519–10529. 31126984 10.1074/jbc.RA119.009199 PMC6615696

[21] Nemčovičová I , Benedict CA and Zajonc DM (2013) Structure of human cytomegalovirus UL141 binding to TRAIL‐R2 reveals novel, non‐canonical death receptor interactions. PLoS Pathog 9, e1003224. 23555243 10.1371/journal.ppat.1003224 PMC3605307

[22] Nemčovičová I , Nemčovič M , Sestak S , Plskova M , Wilson IB and Mucha J (2012) Expression, purification and preliminary crystallographic analysis of drosophila melanogaster lysosomal alpha‐mannosidase. Acta Crystallogr Sect F Struct Biol Cryst Commun 68, 965–970. 10.1107/S1744309112029375 PMC3412785 22869134

[23] Nemčovičová I and Zajonc DM (2014) The structure of cytomegalovirus immune modulator UL141 highlights structural Ig‐fold versatility for receptor binding. Acta Crystallogr D Biol Crystallogr 70, 851–862. 24598754 10.1107/S1399004713033750 PMC3949518

[24] Šedý JR , Balmert MO , Ware BC , Smith W , Nemčovičová I , Norris PS , Miller BR , Aivazian D and Ware CF (2017) A herpesvirus entry mediator mutein with selective agonist action for the inhibitory receptor B and T lymphocyte attenuator. J Biol Chem 292, 21060–21070. 29061848 10.1074/jbc.M117.813295 PMC5743079

[25] Lenhartová S , Nemčovič M , Šebová R , Benko M , Zajonc D and Nemčovičová I (2021) Molecular characterization of the native (non‐linked) CD160–HVEM protein complex revealed by initial crystallographic analysis. Crystals 11, 14.

[26] Benko M and Nemčovičová I (2020) Biological and binding properties of the clinically significant human cytomegalovirus UL144 glycoprotein (dissertation thesis). Univerzita Komenského v Bratislave, Prírodovedecká Fakulta, Bratislava, 126

[27] Lenhartová S and Nemčovičová I (2021) The molecular and structural characterization of viral glycoprotein UL144 (humanus vs. simia) and its binding to human cell receptor CD160 (dissertation thesis). Univerzita Komenského v Bratislave, Prírodovedecká Fakulta, Bratislava, 148

[28] Zhao Y , Chapman DA and Jones IM (2003) Improving baculovirus recombination. Nucleic Acids Res 31, e6. 12527795 10.1093/nar/gng006 PMC140531

